# Identification of candidate genes for drought tolerance in soybean through QTL mapping and gene expression analysis

**DOI:** 10.3389/fgene.2025.1564160

**Published:** 2025-03-26

**Authors:** Gi-Rim Park, Seon-Hwa Bae, Beom-Kyu Kang, Jeong-Hyun Seo, Jae-Hyeon Oh

**Affiliations:** ^1^ Upland Crop Breeding Research Division, National Institute of Crop Science, Rural Development Administration, Miryang-si, Gyeongnam, Republic of Korea; ^2^ Fruit Research Division, National Institute of Horticultural and Herbal Science, Rural Development Administration, Iseo-myeon, Wanju-gun, Republic of Korea; ^3^ Gene Engineering Division, National Institute of Agricultural Sciences, Rural Development Administration, Jeonju-si, Jeollabuk-do, Republic of Korea

**Keywords:** soybean, drought tolerance, QTL, RNA sequencing, candidate genes

## Abstract

**Introduction:**

Drought stress significantly reduces soybean yield, underscoring the need to develop drought-resistant varieties and identify the underlying genetic mechanisms. However, the specific genes and pathways contributing to drought tolerance remain poorly understood. This study aimed to identify candidate genes associated with drought tolerance in soybean using a recombinant inbred line (RIL) population derived from PI416937 and Cheongsang.

**Methods:**

A quantitative trait loci (QTL) mapping study using a 180K high-quality SNP array and composite interval mapping on 140 recombinant inbred lines, coupled with RNA sequencing of treated and control groups, was conducted to identify candidate genes for drought tolerance in soybean.

**Results and Discussion:**

Through QTL mapping and differential gene expression profiling, five candidate genes were identified, with two (*Glyma.06G076100* and *Glyma.10G029600*) highlighted as putative candidates based on functional annotations. These genes appear to play critical roles in stress tolerance, including ion homeostasis and the regulation of plasma membrane ATPase, as well as the synthesis of heat shock proteins (HSPs) that mitigate dehydration and thermal stress. These findings advance our understanding of the genetic basis of drought tolerance in soybean and provide valuable targets for breeding programs aimed at developing resilient cultivars.

## 1 Introduction

Drought stress is a major abiotic factor that significantly reduces soybean yield, with losses exceeding 50% in severe cases (Wang et al., 2003). Combined with flooding, drought contributes to 40%–60% of global yield loss in soybeans and other major crops, including corn, rice, and wheat (Valliyodan and Nguyen, 2006; Krasensky and Jonak, 2012; Ahmed et al., 2013). With the increasing frequency and intensity of droughts due to climate change, developing drought-tolerant soybean cultivars is critical to ensuring food security and agricultural sustainability.

Drought tolerance is a complex trait regulated by networks of genes that control key physiological processes such as water uptake, osmotic adjustment, stomatal regulation, and stress signaling (Harb et al., 2010; Agurla et al., 2018; Abhilasha and Roy Choudhury, 2021; Haghpanah et al., 2024). These processes are influenced by genetic and environmental factors, complicating the identification of stable and reliable markers for drought tolerance (Dhungana et al., 2021; Haghpanah et al., 2024; Saeed et al., 2025). Soybean cultivars exhibit considerable genetic diversity in their ability to tolerate water stress, making breeding programs increasingly focused on specific traits associated with drought tolerance. Key physiological traits linked to drought tolerance include root system architecture, osmotic adjustment, stomatal regulation, and leaf area reduction. For instance, the drought-tolerant accession PI416937 is notable for its fibrous root system, which enables deeper soil water access and enhances drought resilience compared to other cultivars (Fallen et al., 2023; Abdel-Haleem et al., 2011; Gilbert et al., 2011). Extensive research on this accession has identified several QTLs associated with root structure, osmotic regulation, and slow wilting (Abdel-Haleem et al., 2011; Carpentieri-Pipolo et al., 2012; Ye et al., 2020; Kwon et al., 2024).

Drought tolerance is a polygenic trait influenced by multiple genes and environmental factors, making it challenging to evaluate based on a single trait. To address this complexity, in both our previous and current studies, we calculate weighted drought coefficient (WDC) values through statistical analysis of vegetative and reproductive traits. These traits include plant height (PH), number of nodes on the main stem (NN), number of branches (BN), number of pods (PN), biomass (BM), and leaf area (LA), all of which are highly sensitive to drought stress. This approach aims to provide a holistic understanding of the genetic basis of drought tolerance in soybean.

Advances in molecular breeding and next-generation sequencing technologies have facilitated the identification of quantitative trait loci (QTLs) linked to agronomically important traits (Rasheed et al., 2022). Identified QTLs, such as qSWPP19 and qSWPP17 for seed weight (Ren et al., 2020), qPH7-4 and qPH7-6 for plant height (Zhang et al., 2022), qCT3 for canopy temperature regulation (Bazzer and Purcell, 2020), and qSW for slow wilting (Ye et al., 2020; Kwon et al., 2024), are pivotal to drought tolerance. These discoveries, enabled by recombinant inbred line (RIL) analysis and genome-wide association studies (GWAS), provide valuable tools for marker-assisted selection (MAS) and QTL pyramiding to improve drought resilience in soybean cultivars (Rasheed et al., 2022). However, the genetic basis of drought tolerance remains highly complex, involving numerous interacting genes that complicate the effective integration of QTLs into breeding programs.

Recent advancements in genomic technologies, including high-density SNP genotyping, have enhanced the precision of QTL and gene identification associated with drought tolerance, accelerating the development of resilient cultivars. Transcriptomic approaches, such as RNA sequencing (RNA-seq), have advanced our understanding by enabling detailed analysis of gene expression changes under drought stress (Liu et al., 2015; Fracasso et al., 2016; Zhao et al., 2020; Dhungana et al., 2021; Aleem et al., 2021; Chu et al., 2021; Yang et al., 2023). These technologies provide insights into the regulatory networks governing drought tolerance. They demonstrate how genetic variation in drought-responsive genes—involved in water use efficiency, stomatal regulation, and cellular protection—contributes to increased resilience. High-throughput genome and transcriptome sequencing has also facilitated the identification of transcriptional regulators and stress-responsive genes in soybean. Prominent transcription factor families, including Dehydration-Responsive Element Binding (DREB), NAC, WRKY, MYB, and AREB (ABA-Responsive Element Binding), play crucial roles in activating stress-responsive pathways and mediating plant responses to environmental stress (Chen et al., 2007; Fuganti-Pagliarini et al., 2017; Nguyen et al., 2019; Wei et al., 2019; Zhou et al., 2020). These transcription factors regulate processes such as osmoregulation and antioxidant defense. However, the stability of candidate genes across different environmental conditions remains variable, highlighting the complexity of drought tolerance as a quantitative trait. Further research is needed to discover and validate genomic regions associated with drought tolerance, which will provide deeper insights into its mechanisms and practical applications for crop improvement.

Despite progress in identifying QTLs and candidate genes for drought tolerance in soybean, the genetic basis of this trait remains poorly understood due to its complexity and environmental variability. To address these gaps, this study investigates the genetic architecture of drought tolerance using a recombinant inbred line (RIL) population derived from a cross between PI416937, a drought-tolerant accession, and Cheonsang, a drought-sensitive cultivar. By integrating QTL mapping, RNA-seq analysis, and ortholog analysis based on Arabidopsis genes, we aim to identify key loci and genes that contribute to drought resilience. Our findings are expected to provide novel insights into the genetic mechanisms underlying drought tolerance, offering valuable targets for soybean breeding programs.

## 2 Materials and methods

### 2.1 Plant material

An RIL population consisting of 140 lines, advanced to the F8 generation through selfing, was developed from a cross between the drought-tolerant accession PI416937 and the drought-sensitive cultivar Cheonsang. This population was used to analyze genomic regions associated with drought tolerance. The parents and RIL population were cultivated in a greenhouse at the National Institute of Crop Science, Rural Development Administration, Miryang (N35°29′32″ E128°44′35″) from 2017 to 2019. Seeds were sown with a spacing of 25 cm between plants and 30 cm between rows. Irrigation was provided via a drip hose system. To simulate drought conditions, irrigation was discontinued at the V4-R4 stage in the drought treatment plots, whereas it was maintained in the control plots. The experiments were organized into two blocks, each containing two replicates. Soil moisture content in control and drought-stressed plots was measured using a TDR 300 soil moisture meter (Sperctrum Technologies, Plainfield, IL, United States).

### 2.2 Phenotyping and WDC analysis

Phenotyping was conducted for parental lines and 140 RILs across eight traits: PH, number of nodes (ND), number of branches (BR), number of pods (PD), biomass (BM), LA, 100-seed-weight (SW), and seed yield (YD). LA was measured at the R3-R4 stage on the 3^rd^ node below the fresh trifoliate leaves at the apex. PH, nodes (ND), branches (BR), and pods (PD) were measured at the R6 stage, while biomass (BM), 100-SW, and seed yield (YD) were measured at harvest (R8 stage). The drought coefficient (DC) for each trait was calculated as the ratio of the trait value under drought conditions to that under control conditions, using the equation:

DC=Trait Drought/Trait Control

The WDC was statistically calculated based on the eight traits using the formula described by Dhungana et al. (2021). Phenotypic data collected from 2017 to 2019 for the parental lines and 140 RILs were combined. Traits were grouped into combinations of 2, 3, 5, 6, and 8 for WDC calculation, resulting in 22 drought coefficients that reflected the years of investigation and phenotype combinations. These coefficients were utilized for QTL analysis.

$$WDC=\sum_{i=1}^{n}\left[DC\times \left(\left|ri\right|÷\sum_{i=1}^{n}\left|ri\right|\right)\right]$$

### 2.3 Genotyping of the RIL population

Genomic DNA was extracted from fresh trifoliate leaves using an Exgene Plant SV Miniprep kit (GeneAll, Seoul, Korea), following the manufacturer’s instructions. The parents and RILs were genotyped using the 180K Axiom® Soya SNP array (Lee et al., 2015) to construct a high-density linkage map.

### 2.4 Construction of linkage map and QTL analysis

Polymorphic markers between the parents were selected from the 180K SNP dataset and analyzed for redundancy. Redundant markers with identical segregation patterns and clustered at the same genetic positions were removed using the Bin function in ICIMapping version 4.1 (Meng et al., 2015). During this process, markers with significant segregation distortion (p < 0.05) and a missing rate exceeding 5% were excluded. The linkage map was constructed using the Kosambi mapping function, with adjusted parameters including a grouping LOD threshold of 3.0, marker ordering by nnTwoOpt, and rippling based on adjacent recombination fractions.

QTLs for drought tolerance were identified using composite interval mapping (CIM) in QTL Cartographer version 2.5 (available at https://statgen.ncsu.edu/qtlcart). The CIM analysis was performed with Model 6, incorporating forward and backward regression, a scanning interval of 1.0 cM between markers, and a window size of 10 cM. The LOD threshold for each trait was determined using 1,000 permutation tests at *p* < 0.05. QTLs were named according to the method described by Dhungana et al. (2020).

### 2.5 Prediction of candidate genes through ortholog analysis

Candidate genes within QTL regions were queried using the National Center for Biotechnology Information database, Kyoto Encyclopedia of Genes and Genomes Pathway Database (www.genome.jp/kegg/pathway.html), and Phytozome (www.phytozome.net) to analyze sequence similarity based on orthologs. Comparative analyses were conducted using BLASTX with an e-value threshold of 1e-10, a minimum sequence identity of 40%, and a query coverage of at least 40%. Soybean genes aligning with *Arabidopsis thaliana* genes were identified and prioritized for further analysis.

### 2.6 RNA sequencing and transcriptome analysis

Two parental lines of the RIL population, representing drought tolerance and susceptibility, were selected along with two RILs showing high tolerance and high susceptibility to drought. To analyze differences in gene expression levels, both treated and control groups were used. RNA was extracted from three plants of each line, and six samples per line (three replicates for each group) were analyzed, resulting in a total of 36 samples (Table 1). Total RNA was isolated from frozen leaf samples using a Qiagen RNA isolation kit, and mRNA was converted into library templates using the TruSeq RNA Sample Prep Kit v2 (Illumina, Cat No. 15026495 Rev. F). Library preparation included mRNA purification, strand synthesis, end repair, adapter ligation, and PCR enrichment. Enriched libraries were quantified and sequenced using the Illumina HiSeq 4000 platform.

**TABLE 1:** Parental lines and recombinant inbred lines used for transcriptome analysis.

	Parental line		RIL population			
	PI416937 (Drought-tolerant)	Cheonsang (Drought-sensitive)	Drought-tolerant		Drought-sensitive	
			19T52056	19T52084	19T52018	19T52053
Control	C-1_1	C-2_1	C-56_1	C-84_1	C-18_1	C-53_1
	C-1_2	C-2_2	C-56_2	C-84_2	C-18_2	C-53_2
	C-1_3	C-2_3	C-56_3	C-84_3	C-18_3	C-53_3
Drought treatment	D-1_1	D-2_1	D-56_1	D-84_1	D-18_1	D-53_1
	D-1_2	D-2_2	D-56_2	D-84_2	D-18_2	D-53_2
	D-1_3	D-2_3	D-56_3	D-84_3	D-18_3	D-53_3

The raw sequence in FASTQ format, generated by the Illumina platform, was processed using open-source bioinformatics tools. Quality assessment was performed to calculate base quality scores, and sequences with a quality score (Q) below 15 were trimmed. Adapter sequences were removed using Cutadapt (http://code.google.com/p/cutadapt) (Supplementary Table S1). After trimming, sequences were mapped to the Glycine max Wm82.a2.v1 reference genome using HISAT. Transcription expression levels were quantified by calculating read counts using StringTie (Pertea et al., 2016). Normalization was performed using the DESeq R package (Anders and Huber, 2010) to generate expression values and estimates of variability.

## 3 Results

### 3.1 Phenotypic analysis of parental lines and RIL population

The drought-tolerant parent PI416397 consistently exhibited higher WDC values than the drought-susceptible parent Cheonsang across all trait combinations. The mean WDC values for PI416937, calculated using two, three, and six traits, were 0.75, 0.80, and 0.79, respectively, compared with 0.42, 0.52, and 0.57 for Cheonsang. The WDC distribution of the RIL population over a continuous 3-year period exhibited a normal distribution, suggesting transgressive segregation patterns (Table 2).

**TABLE 2:** Weighted drought coefficient (WDC) of the parental lines and recombinant inbred lines (RILs) for 3 years (2017–2019).

Trait	Year	Parental line		RIL population	
		PI416937	Cheonsang	Mean	Range
WDC (2)	2017	0.71	0.28	0.73	0.12–2.16
	2018	0.65	0.53	0.57	0.23–1.01
	2019	0.9	0.45	0.56	0.23–1.04
	Mean	0.75	0.42	0.62	0.12–1.04
WDC (3)	2017	0.78	0.43	0.77	0.32–1.87
	2018	0.76	0.62	0.65	0.36–0.99
	2019	0.86	0.51	0.62	0.31–1.02
	Mean	0.8	0.52	0.68	0.31–1.87
WDC (6)	2017	0.76	0.45	0.86	0.22–3.06
	2018	0.78	0.63	0.67	0.38–1.01
	2019	0.82	0.62	0.7	0.33–1.15
	Mean	0.79	0.57	0.74	0.22–3.06

1 Average value of three continuous years. The values in the parentheses after WDC, indicate the number of traits considered to calculate WDC: 2 (biomass and leaf area), 3 (plant height, biomass, and leaf area), and 6 (plant height, node number, branch number, pod number, biomass, and leaf area).

### 3.2 Linkage mapping and QTL analysis

**FIGURE 1:**
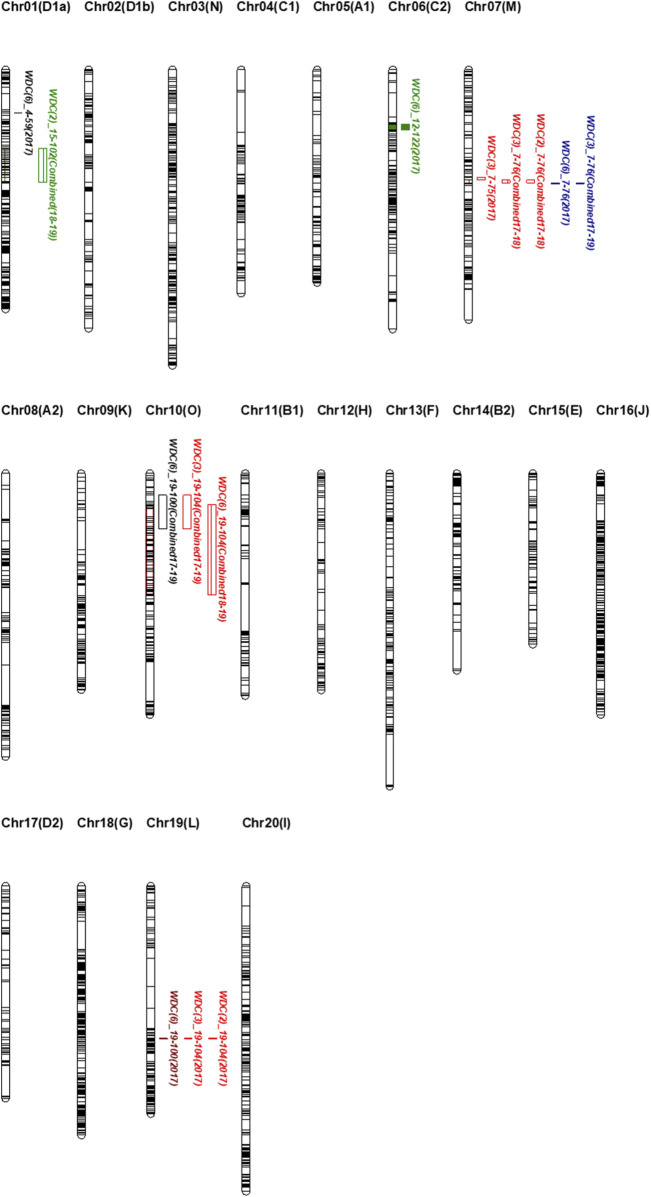
High-density genetic maps for 5 soybean chromosomes (i.e., 1, 6, 7, 10, and 19) developed using the PI416937/Cheonsang RIL population. Shown are QTL locations for all WDC plants.

A total of 2,595 SNPs were used to construct the linkage maps of 20 soybean chromosomes. The total map spanned 3,610.36 cM, with an average marker spacing of 1.39 cM. Chromosome 3 had the longest linkage map at 219.35 cM, whereas Chromosome 5 had the shortest at 110.68 cM (Supplementary Table S2). Nine QTLs (LOD ≥ 3) associated with drought tolerance were identified across nine combinations of WDCs, distributed on Chromosomes 1, 6, 7, 10, and 19. The LOD values of these QTLs ranged from 3.00 to 4.54, and the phenotypic variation explained (PVE) ranged from 6.78% to 14.69% (Table 3). Of the nine QTLs, one was located on chromosomes 6 and 19, two were found on chromosomes 1 and 10, and three were identified on chromosome 7 (Figure 1). Notably, qWDC7-2, qWDC7-3, and qWDC10-1 were commonly detected in two trait combinations (biomass and leaf area), whereas qWDC19-1 was identified in three combinations (PH, biomass, and leaf area), suggesting stability across different trait analyses.

**TABLE 3:** QTLs for drought tolerance identified in a recombination inbred line population derived from a drought-tolerant “PI416937” and susceptible “Cheonsang” cultivar.

QTL	Trait	Year	Chr.	Genetic position (cM)	Marker interval	Physical location (bp)	LOD	PVE (%)	Addictive effect
qWDC1-1	6	2017	1	32	AX-90508127-AX-90328138	2365692-2618112	3.33	6.7817	0.1326
qWDC1-2	2	Mean (2018–2019)	1	55	AX-90462973-AX-90512118	4314553-4439457	3.30	10.3636	0.0379
qWDC6-1	6	2017	6	54	AX-90508939-AX-90432389	5327742-6031694	4.55	9.3407	0.1567
qWDC7-1	3	2017	7	77	AX-90383551-AX-90514687	8566820-9630217	3.00	9.073	−0.0841
qWDC7-2	3	Mean (2017–2018)	7	79	AX-90514687-AX-90450726	9630217-10232736	3.38	10.5322	−0.0475
	2	Mean (2017–2018)		79	AX-90514687-AX-90450726	9630217-10232736	3.29	9.3114	−0.058
qWDC7-3	6	2017	7	80	AX-90450726-AX-90512690	10232736-10403810	4.23	9.0109	−0.1531
	3	Mean (2017–2019)		80	AX-90450726-AX-90512690	10232736-10403810	3.27	9.4604	−0.0326
qWDC10-1	6	Mean (2017–2019)	10	16	AX-90382407-AX-90366226	2504088-2919759	3.39	9.4418	−0.0486
	3	Mean (2017–2019)		16	AX-90382407-AX-90366226	2504088-2919759	3.16	9.5485	−0.0329
qWDC10-2	6	Mean (2017–2019)	10	21	AX-90331361-AX-90353817	2802357-3026040	3.19	9.2411	−0.0296
qWDC19-1	6	2017	19	155	AX-90489570-AX-90486461	42347892-42400298	4.55	9.3451	0.1558
	3	2017		155	AX-90489570-AX-90486461	42347892-42400298	3.79	11.4527	0.0942
	2	2017		155	AX-90489570-AX-90486461	42347892-42400298	4.23	14.6923	0.1371

### 3.3 Identification of genes in QTL regions

**FIGURE 2:**
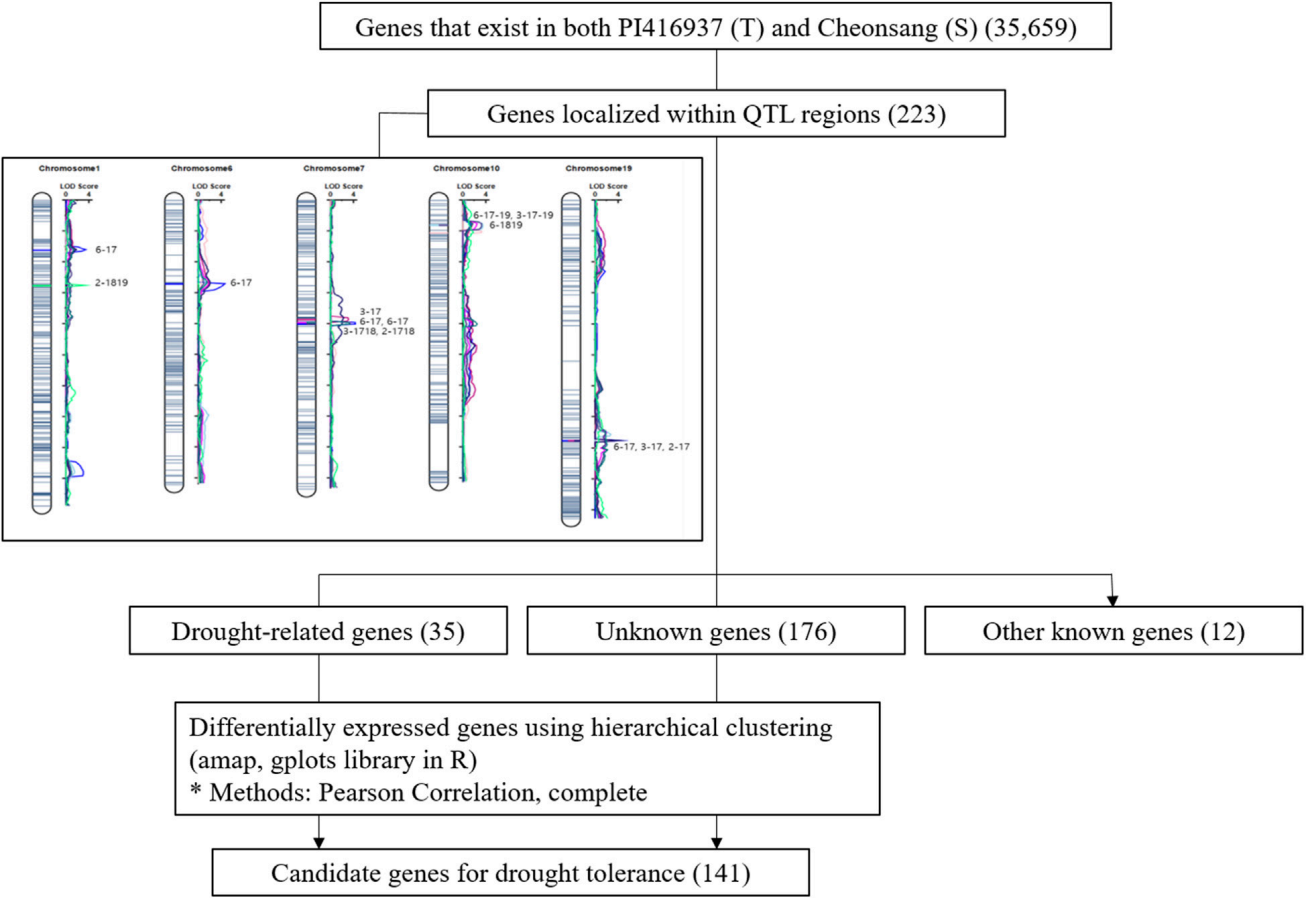
Flow chart of the process used to identify candidate genes related drought tolerance.

A total of 223 genes were identified within the QTL regions, distributed as follows: 25 genes on chromosome 1, 69 genes on chromosome 6, 85 genes on chromosome 7, 39 genes on chromosome 10, and 5 genes on chromosome 19. Of them, 35 genes aligned with drought-related *A. thaliana* genes, including heat shock transcription factors and members of the glutathione S-transferase family. Additionally, 176 genes were annotated as having unknown functions, and 12 were identified with other known functions. The overall analysis workflow used to identify these genes in summarized in Figure 2.

### 3.4 RNA sequencing

For transcriptomic analysis, raw data generated by Illumina sequencing were initially filtered based on the average Phred score value of the sequences, measured in units of 4 bp. Sequences with an average Phred score of 20 or less were removed, resulting in the exclusion of approximately 20% of the raw data. The resulting dataset included paired sequences with a minimum fragment length of 36 bp. Sequence alignment was performed using *Glycine* max Wm82.a2.v1 as the reference genome, achieving an average mapping rate of 87.8% (Supplementary Table S3).

### 3.5 DEG analysis

Transcript expression levels were quantified as read counts using StringTie2. For DEG analysis in DES eq 2, 3 biological replicates within the control and treatment groups were averaged, resulting in 12 samples analyzed in total. Of the 75,384 transcripts identified with read counts greater than or equal to 1, transcripts were considered differentially expressed if their absolute log 2 fold change was ≥ 2 and the adjusted p-value (p-adj) was <0.05. These transcripts were included in downstream analyses. To investigate gene expression changes induced by drought treatment, comparisons were made between drought-treated tolerant lines (dCS, dR56, and dR84) and the PI control lines. A total of 1,826 transcripts showed increased expression in response to drought treatment; however, 38 transcripts were excluded because their expression levels also increased in drought-treated susceptible lines.

### 3.6 Clustering-based gene expression patterns

**FIGURE 3:**
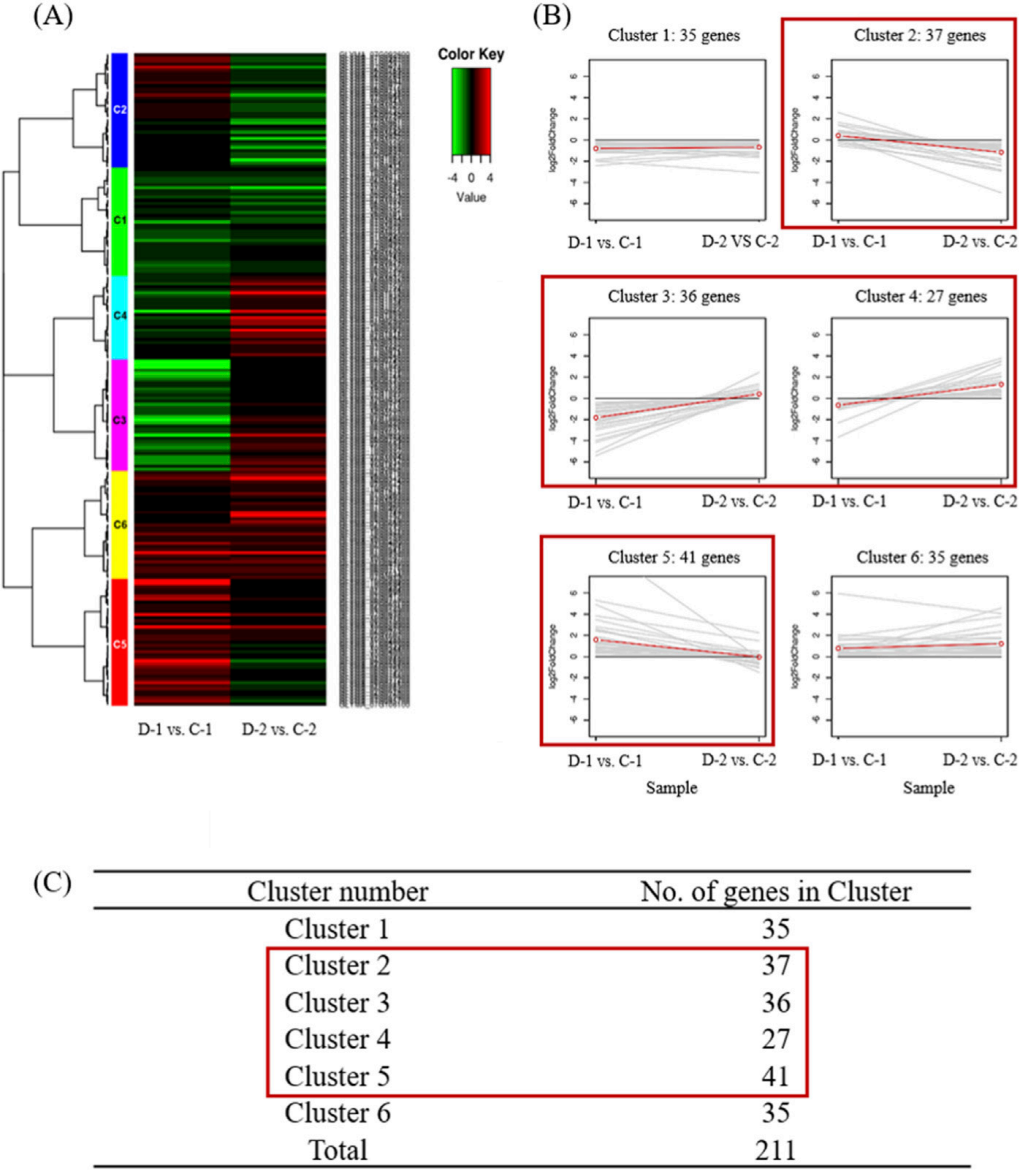
Clustering analysis of gene expression patterns in sensitivity and drought-tolerant cultivars using 211 genes. **(A)** Differentially expressed gene (DEG) analysis showed six clusters that were differentially expressed in D-1 vs C-1 and D-2 vs C-2 samples. Gene expression profiles of D-1 vs C-1 and D-2 vs C-2 combinations. **(B)** Line plots of clusters. Expression patterns of genes in the six clusters, namely, Cluster 1–Cluster 6, corresponding to the hierarchical heatmap. Red boxes indicate clusters that showed significant differential expression. **(C)** Number of genes in the clustering set; 141 candidate genes were found in Clusters 2 to 5.

To identify phenotype-associated genes located within the QTL regions, clustering was performed on RNA expression data for 211 genes, comprising 35 drought-related genes identified through ortholog analysis and 176 genes with unknown functions. Prior to clustering, raw read counts were normalized using the variance-stabilizing transformation method in DESeq2 to ensure comparability across samples. Hierarchical clustering was conducted based on Person correlation distance and complete linkage to group genes with similar expression patterns. The clustering results were visualized using a heatmap (Figure 3), providing insights into gene expression differences between drought-sensitive and drought-tolerant samples. A total of 141 genes were found to belong to cluster C2-C5, exhibiting distinct expression patterns between drought-sensitive and drought-tolerant samples. These genes were subsequently selected as candidate genes for drought tolerance (Figure 3).

### 3.7 List of putative candidate genes based on ortholog analysis and RNA-Seq

Through QTL analysis, a total of 223 genes were identified within the QTL regions. Of them, 141 genes exhibited differential expression between drought-tolerant and drought-sensitive lines, as confirmed by clustering analysis. To refine the list of candidate genes, we prioritized those with increased expression in tolerant lines and decreased expression in susceptible lines under drought conditions. Among the 141 genes, 47 showed differential expression patterns between the lines (Supplementary Table S4). Based on normalized counts and functional annotation, five putative candidate genes were ultimately selected (Table 4). These genes are located on chromosomes 1, 6, 7, and 10. Two genes, *Glyma.10G029400* and *Glyma.10G029600*, are situated within the *qWDC10-1* region, a stable QTL detected across multiple trait combinations. Additionally, *Glyma.06G076100* and *Glyma.10G029600* were identified with plasma membrane ATPase 4 and heat stress transcription factor *Hsf-21*, respectively.

**TABLE 4:** List of putative candidate genes based on QTL analysis and RNA-seq.

QTL information	Gene ID	Physical location (bp)	Gene description
qWDC1-2	*Glyma.01G040700*	Chr1:4392159-4393458	Uncharacterized LOC100527298
qWDC6-1	*Glyma.06G076100*	Chr6:5893905-5901500	Plasma membrane ATPase 4
qWDC7-1	*Glyma.07G097900*	Chr7:9199620-9202160	Uncharacterized LOC100808843
qWDC10-1	*Glyma.10G029400*	Chr10:2560235-2562772	Uncharacterized LOC100527528
qWDC10-1	*Glyma.10G029600*	Chr10:2575885-2578579	Heat stress transcription factor Hsf-21

## 4 Discussion

Drought tolerance is a complex trait influenced by both genetic and environmental factors, making it challenging to identify key genes involved in this process. Recent advancements in genomic technologies, such as high-density SNP genotyping and RNA-seq, have significantly improved our ability to identify QTLs and gene expression changes associated with drought tolerance in crops like soybean (Waheed et al., 2021; Ouyang et al., 2022; Manjunath et al., 2024). These advancements provide insights into the genetic basis of drought tolerance and are crucial for developing resilient crop varieties. In this study, we utilized the 180K SNP chip, which offers high resolution and accuracy, thereby improving data reliability. Moreover, a WDC, which integrates multiple drought-related traits with assigned weights, was used for phenotypic evaluation to capture a more comprehensive response to drought stress.

In previous studies, over 30 QTLs related to drought tolerance in soybean have been identified on chromosomes 1, 2, 4, 7, 10, and 12. These QTLs have been associated with traits such as slow canopy wilting, root thickness, and drought susceptibility index (Du et al., 2009; Brensha et al., 2012; Ye et al., 2020; Arya et al., 2021). For instance, slow canopy wilting has been extensively studied using drought-tolerant lines such as PI416937, PI567690, and PI567331. Several QTLs have been mapped for this trait, including stable loci on chromosomes 6 and 10 (*qSW_Gm06* and *qSW_Gm10*), which account for 20%–30% of the phenotypic variation across diverse environmental conditions (Ye et al., 2020; Rasheed et al., 2022).

In this study, 9 QTLs associated with drought tolerance were identified on chromosomes 1, 6, 7, 10, and 19. Notably, the QTLs identified on chromosomes 6 and 10, as reported in previous findings (Ye et al., 2020; Kwon et al., 2024), suggest their consistent role in conferring drought tolerance. A total of 223 genes were initially mapped within these QTL regions, of which 141 displayed differential expression between drought-tolerant and susceptible plant lines. Among these 141 genes showing differential expression, 47 exhibited significant expression changes under drought stress, and five were selected as putative candidates based on their expression patterns and functional annotations. These candidate genes, located on chromosomes 1, 6, 7, and 10, reside in stable QTL regions consistently linked to drought tolerance across various environmental conditions.

According to Dhungana et al. (2021), QTL analysis using the same segregating population and phenotypic evaluation methods identified genomic regions on five chromosomes: 2, 7, 10, 15, and 20. Chromosomes 7 and 10, in particular, showed consistent results with those of previous studies. Differences in identified QTL regions may result from variations in SNP filtering criteria. Nonetheless, the regions on chromosomes 7 and 10, common to both analyses, likely play a significant role in drought tolerance. In this study, a distinct strategy was adopted to select candidate genes strongly associated with drought tolerance traits. Instead of following the approach of previous studies, we focused on genes located within QTL regions and explored their expression differences in tolerant and susceptible lines before and after drought treatment.

A comparison of gene expression levels revealed that the drought-tolerant accession exhibited a significant upregulation of stress-responsive genes, with an average fold change exceeding 3.8-fold. In contrast, the drought-sensitive accession showed a decrease in gene expression, with an average reduction of 0.4-fold, highlighting its limited transcriptional response to drought stress. Ortholog analysis identified two candidate genes, *Glyma.06G076100* and *Glyma.10G029600*, which are closely associated with drought-related genes in *A. thaliana*. *Glyma.06G076100*, annotated as a Plasma Membrane ATPase 4 (PMA4), plays a crucial role in maintaining ion homeostasis and osmotic balance under stress conditions by regulating ATPase activity in the plasma membrane (Baxter et al., 2003). This gene has been shown to enhance plant stress tolerance by facilitating the management of ionic gradients and maintaining cellular functions during environmental stress (Gévaudant et al., 2007; Li et al., 2022). Similar findings in other crops, such as rice, have confirmed the role of PMA4 and its orthologs in drought tolerance (Duby and Boutry, 2009; Zhang et al., 2013), supporting its potential for improving drought resilience in soybean. Furthermore, although located in a genomic region distinct from this study, *Glyma.10g019000*—a key gene associated with ATPase activity within the *qSW_Gm10* region—has been implicated in drought tolerance through the regulation of stomatal opening (Zhao et al., 2019; Kwon et al., 2024).

*Glyma.10g029600* may contribute to drought tolerance through the synthesis of stress-related proteins, particularly heat shock proteins (HSPs). These proteins are crucial for maintaining proper protein folding and cellular integrity during stress (Ahuja et al., 2010; Tiwari et al., 2015; Bakthisaran et al., 2015; Ul Haq et al., 2019). HSPs protect plant cells from dehydration and thermal stress by preventing protein aggregation and ensuring proper protein functionality (Ul Haq et al., 2019; Ding et al., 2023). The upregulation of genes encoding HSPs under drought conditions suggests that *Glyma.10g29600* may play a role in these protective mechanisms, possibly by regulating osmotic balance and stabilizing cellular structures. In soybean, heat stress transcription factor (Hsf) regulates plant responses to multiple abiotic stresses, including heat, drought, and salinity (Li et al., 2014; Guo et al., 2016; Wang et al., 2021). Hsf activates stress-responsive genes, underscoring its importance in mitigating the adverse effects of environmental stresses (Joshi et al., 2016; Guo et al., 2016; Song et al., 2017; Hussain et al., 2021).

The identification of *Glyma.06G076100* and *Glyma.10G029600* as key candidate genes highlights their potential utility in breeding programs. For example, PMA4 has been implicated in maintaining ion homeostasis, a critical component of drought resilience, while HSPs play a pivotal role in stabilizing cellular structures under stress. Incorporating these genes into MAS frameworks could accelerate the development of drought-tolerant soybean cultivars. Furthermore, exploring the expression patterns of novel genes identified in this study may uncover previously uncharacterized pathways involved in drought response. These findings underscore the importance of integrating QTL mapping, transcriptomic analysis, and functional genomics to address the growing challenges of climate change and ensure agricultural sustainability.

In addition to these well-characterized genes, other genes within the identified QTL regions exhibited significant differential expression between drought-tolerant and drought-sensitive accessions. These genes may represent novel candidates for drought tolerance that remain unexplored. While their precise roles in stress response mechanisms remain unclear, their differential expression patterns suggest involvement in physiological processes contributing to drought tolerance. Further functional validation of these genes is essential to elucidate their specific roles and evaluate their potential as targets for breeding drought-tolerant soybean cultivars.

## Data Availability

The datasets presented in this study can be found in online repositories. The names of the repository/repositories and accession number(s) can be found in the article/Supplementary Material.
