# Supplementary material for: Identification of candidate genes for drought tolerance in soybean through QTL mapping and gene expression analysis
Source: Front Genet. 2025 Mar 26;16:1564160. doi: 10.3389/fgene.2025.1564160 (PMC11980780; doi:10.3389/fgene.2025.1564160)
Supplement: Supplementary file 1 [file Supplementaryfile1.docx]

Supplementary Material

# Supplementary Tables

**Table S1.** Trimmed RNA-seq data.

| Sample | Raw data | | Trimmed data | | Rate |
| --- | --- | --- | --- | --- | --- |
|  | Num_seqs^1^ | Sum_len^2^ | Num_seqs | Sum_len |  |
| C-1_1 | 74,095,158 | 7,483,610,958 | 63,865,146 | 6,257,033,971 | 83.6% |
| C-1_2 | 57,539,776 | 5,811,517,376 | 48,221,930 | 4,705,483,269 | 81.0% |
| C-1_3 | 59,433,768 | 6,002,810,568 | 49,103,810 | 4,782,554,203 | 79.7% |
| C-18_1 | 73,711,780 | 7,444,889,780 | 63,398,274 | 6,208,399,436 | 83.4% |
| C-18_2 | 53,087,094 | 5,361,796,494 | 44,242,982 | 4,310,203,221 | 80.4% |
| C-18_3 | 58,115,296 | 5,869,644,896 | 47,521,426 | 4,621,245,794 | 78.7% |
| C-2_1 | 73,069,368 | 7,380,006,168 | 63,009,656 | 6,174,340,130 | 83.7% |
| C-2_2 | 73,290,246 | 7,402,314,846 | 60,735,914 | 5,917,958,395 | 79.9% |
| C-2_3 | 72,520,720 | 7,324,592,720 | 60,957,210 | 5,944,909,591 | 81.2% |
| C-53_1 | 67,242,692 | 6,791,511,892 | 56,041,384 | 5,455,353,616 | 80.3% |
| C-53_2 | 54,640,636 | 5,518,704,236 | 45,320,138 | 4,415,923,917 | 80.0% |
| C-53_3 | 70,310,714 | 7,101,382,114 | 58,893,804 | 5,742,201,756 | 80.9% |
| C-56_1 | 73,507,712 | 7,424,278,912 | 62,364,772 | 6,096,475,541 | 82.1% |
| C-56_2 | 71,348,818 | 7,206,230,618 | 59,075,010 | 5,752,486,990 | 79.8% |
| C-56_3 | 54,173,378 | 5,471,511,178 | 45,037,926 | 4,393,856,756 | 80.3% |
| C-84_1 | 73,748,476 | 7,448,596,076 | 63,341,442 | 6,200,913,075 | 83.2% |
| C-84_2 | 68,819,682 | 6,950,787,882 | 57,459,264 | 5,599,202,128 | 80.6% |
| C-84_3 | 69,920,326 | 7,061,952,926 | 58,658,718 | 5,727,234,333 | 81.1% |
| D-1_1 | 72,472,908 | 7,319,763,708 | 61,469,738 | 5,998,577,283 | 82.0% |
| D-1_2 | 56,514,096 | 5,707,923,696 | 45,672,470 | 4,428,347,922 | 77.6% |
| D-1_3 | 56,046,592 | 5,660,705,792 | 45,961,090 | 4,469,063,014 | 78.9% |
| D-18_1 | 71,953,306 | 7,267,283,906 | 60,639,234 | 5,921,046,990 | 81.5% |
| D-18_2 | 65,009,676 | 6,565,977,276 | 53,751,222 | 5,235,840,569 | 79.7% |
| D-18_3 | 51,123,000 | 5,163,423,000 | 42,496,060 | 4,138,474,614 | 80.1% |
| D-2_1 | 71,398,378 | 7,211,236,178 | 61,587,312 | 6,038,897,503 | 83.7% |
| D-2_2 | 67,883,502 | 6,856,233,702 | 56,754,034 | 5,537,356,784 | 80.8% |
| D-2_3 | 52,677,830 | 5,320,460,830 | 42,599,404 | 4,138,128,190 | 77.8% |
| D-53_1 | 73,223,448 | 7,395,568,248 | 62,028,328 | 6,060,234,163 | 81.9% |
| D-53_2 | 66,330,584 | 6,699,388,984 | 54,575,270 | 5,305,305,688 | 79.2% |
| D-53_3 | 55,925,432 | 5,648,468,632 | 45,959,698 | 4,470,095,084 | 79.1% |
| D-56_1 | 71,953,388 | 7,267,292,188 | 60,089,272 | 5,853,200,500 | 80.5% |
| D-56_2 | 54,642,492 | 5,518,891,692 | 44,244,600 | 4,289,515,918 | 77.7% |
| D-56_3 | 69,957,098 | 7,065,666,898 | 56,769,104 | 5,512,220,333 | 78.0% |
| D-84_1 | 72,685,690 | 7,341,254,690 | 61,053,084 | 5,954,014,612 | 81.1% |
| D-84_2 | 74,853,754 | 7,560,229,154 | 61,308,262 | 5,958,144,444 | 78.8% |
| D-84_3 | 80,130,292 | 8,093,159,492 | 65,493,742 | 6,343,423,496 | 78.4% |

^1^ Num_seqs: number of sequences

^2^ Sum_len: total length.

**Table S2.** Statistics for the genetic map constructed using F2 populations of a cross between the “PI416937” and “Cheonsang” soybean cultivars.

| Chromosome | No. of Markers | Length, cM |
| --- | --- | --- |
| Chromosome1 | 159 | 197.25 |
| Chromosome2 | 151 | 211.65 |
| Chromosome3 | 174 | 219.35 |
| Chromosome4 | 98 | 177.83 |
| Chromosome5 | 74 | 110.68 |
| Chromosome6 | 122 | 183.8 |
| Chromosome7 | 122 | 186.62 |
| Chromosome8 | 102 | 157.92 |
| Chromosome9 | 175 | 182.43 |
| Chromosome10 | 114 | 182.43 |
| Chromosome11 | 86 | 145.88 |
| Chromosome12 | 95 | 190.59 |
| Chromosome13 | 192 | 213.31 |
| Chromosome14 | 109 | 149.31 |
| Chromosome15 | 59 | 120.67 |
| Chromosome16 | 207 | 194.73 |
| Chromosome17 | 100 | 196.32 |
| Chromosome18 | 191 | 203.26 |
| Chromosome19 | 122 | 205.24 |
| Chromosome20 | 143 | 181.09 |
| Total | 2,595 | 3,610.36 |

**Table S3.** Mapped RNA-seq results.

| Sample | Trimmed sequences | Mapped and paired reads | Mapping rate |
| --- | --- | --- | --- |
| C-1_1 | 63,865,146 | 54,462,324 | 85.3% |
| C-1_2 | 48,221,930 | 40,744,498 | 84.5% |
| C-1_3 | 49,103,810 | 41,633,466 | 84.8% |
| C-18_1 | 63,398,274 | 58,321,082 | 92.0% |
| C-18_2 | 44,242,982 | 40,703,890 | 92.0% |
| C-18_3 | 47,521,426 | 43,651,346 | 91.9% |
| C-2_1 | 63,009,656 | 57,972,700 | 92.0% |
| C-2_2 | 60,735,914 | 53,559,062 | 88.2% |
| C-2_3 | 60,957,210 | 53,666,466 | 88.0% |
| C-53_1 | 56,041,384 | 51,337,368 | 91.6% |
| C-53_2 | 45,320,138 | 38,021,988 | 83.9% |
| C-53_3 | 58,893,804 | 49,842,260 | 84.6% |
| C-56_1 | 62,364,772 | 59,119,712 | 94.8% |
| C-56_2 | 59,075,010 | 55,590,982 | 94.1% |
| C-56_3 | 45,037,926 | 42,462,580 | 94.3% |
| C-84_1 | 63,341,442 | 60,205,568 | 95.0% |
| C-84_2 | 57,459,264 | 53,899,114 | 93.8% |
| C-84_3 | 58,658,718 | 54,919,600 | 93.6% |
| D-1_1 | 61,469,738 | 54,554,710 | 88.8% |
| D-1_2 | 45,672,470 | 40,802,588 | 89.3% |
| D-1_3 | 45,961,090 | 40,641,424 | 88.4% |
| D-18_1 | 60,639,234 | 53,011,750 | 87.4% |
| D-18_2 | 53,751,222 | 46,844,288 | 87.2% |
| D-18_3 | 42,496,060 | 37,062,278 | 87.2% |
| D-2_1 | 61,587,312 | 50,233,322 | 81.6% |
| D-2_2 | 56,754,034 | 48,540,012 | 85.5% |
| D-2_3 | 42,599,404 | 36,169,882 | 84.9% |
| D-53_1 | 62,028,328 | 51,819,506 | 83.5% |
| D-53_2 | 54,575,270 | 42,537,894 | 77.9% |
| D-53_3 | 45,959,698 | 35,842,584 | 78.0% |
| D-56_1 | 60,089,272 | 49,928,486 | 83.1% |
| D-56_2 | 44,244,600 | 35,136,614 | 79.4% |
| D-56_3 | 56,769,104 | 45,435,424 | 80.0% |
| D-84_1 | 61,053,084 | 55,121,898 | 90.3% |
| D-84_2 | 61,308,262 | 55,820,346 | 91.0% |
| D-84_3 | 65,493,742 | 59,596,916 | 91.0% |

Table S4. List of 47 Genes Showing Differential Expression Patterns Under Drought Stress

| Gene ID | Physical location (bp) | Gene Description |
| --- | --- | --- |
| *Glyma.01G023500* | Chr1:2367321-2373449 | MADS-box protein SVP |
| *Glyma.01G023700* | Chr1:2405107-2414341 | GPI ethanolamine phosphate transferase 2 |
| *Glyma.01G024500* | Chr1:2502596-2504339 | uncharacterized LOC102668650 |
| *Glyma.01G024600* | Chr1:2504633-2507081 | uncharacterized LOC100499731 |
| *Glyma.01G040600* | Chr1:4389420-4392033 | dnaJ homolog subfamily B member 5 |
| *Glyma.01G040700* | Chr1:4392159-4393458 | uncharacterized LOC100527298 |
| *Glyma.01G041200* | Chr1:4420529-4421978 | TRX domain-containing protein |
| *Glyma.06G069700* | Chr6:5345917-5349422 | protein OVEREXPRESSOR OF CATIONIC PEROXIDASE 3 |
| *Glyma.06G071200* | Chr6:5443006-5448211 | boron transporter 4 |
| *Glyma.06G071600* | Chr6:5466908-5468651 | uncharacterized LOC100500647 |
| *Glyma.06G072200* | Chr6:5533994-5547204 | transcriptional corepressor LEUNIG |
| *Glyma.06G072500* | Chr6:5578718-5584118 | coiled-coil domain-containing protein 93 |
| *Glyma.06G073100* | Chr6:5634772-5639307 | E3 ubiquitin-protein ligase At1g63170 |
| *Glyma.06G073200* | Chr6:5644140-5650953 | death-inducer obliterator 1 |
| *Glyma.06G074100* | Chr6:5710970-5712358 | molybdate transporter 1 |
| *Glyma.06G074700* | Chr6:5758402-5786537 | uncharacterized LOC100813647 |
| *Glyma.06G075000* | Chr6:5796960-5805987 | DDB1- and CUL4-associated factor homolog 1 |
| *Glyma.06G076000* | Chr6:5880948-5885101 | COP9 signalosome complex subunit 5a |
| *Glyma.06G076100* | Chr6:5893905-5901500 | plasma membrane ATPase 4 |
| *Glyma.06G076200* | Chr6:5902265-5902594 | uncharacterized LOC102665489 |
| *Glyma.06G076400* | Chr6:5905512-5912377 | uncharacterized LOC100818803 |
| *Glyma.06G076700* | Chr6:5934111-5936867 | uncharacterized protein At1g10890 |
| *Glyma.06G077200* | Chr6:5962956-5964561 | uncharacterized LOC100776044 |
| *Glyma.07G091800* | Chr7:8570088-8572245 | uncharacterized LOC100527326 |
| *Glyma.07G092600* | Chr7:8636143-8643495 | uncharacterized LOC100803163 |
| *Glyma.07G093800* | Chr7:8755633-8760962 | uncharacterized LOC100791882 |
| *Glyma.07G093900* | Chr7:8767005-8771560 | protein SAR DEFICIENT 1 |
| *Glyma.07G094500* | Chr7:8810207-8815766 | PR5-like receptor kinase |
| *Glyma.07G094800* | Chr7:8840028-8840930 | PR5-like receptor kinase |
| *Glyma.07G095100* | Chr7:8864955-8866079 | PR5-like receptor kinase |
| *Glyma.07G095700* | Chr7:8966325-8967389 | LEAF RUST 10 DISEASE-RESISTANCE LOCUS RECEPTOR-LIKE PROTEIN KINASE-like 2.5 |
| *Glyma.07G095800* | Chr7:8969502-8976133 | PR5-like receptor kinase |
| *Glyma.07G096400* | Chr7:9042279-9047301 | uncharacterized LOC100802473 |
| *Glyma.07G097400* | Chr7:9141472-9143551 | uncharacterized LOC102668299 |
| *Glyma.07G097900* | Chr7:9199620-9202160 | uncharacterized LOC100808843 |
| *Glyma.07G099100* | Chr7:9347781-9349110 | protein argonaute 4 |
| *Glyma.07G099400* | Chr7:9398756-9411345 | nuclear poly(A) polymerase 1 |
| *Glyma.07G099700* | Chr7:9450842-9454377 | uncharacterized LOC106799226 |
| *Glyma.07G103000* | Chr7:9849051-9873480 | uncharacterized LOC100779987 |
| *Glyma.07G106000* | Chr7:10281063-10282376 | U-box domain-containing protein 21 |
| *Glyma.07G106100* | Chr7:10292226-10305231 | extra-large guanine nucleotide-binding protein 3 |
| *Glyma.10G029400* | Chr10:2560235-2562772 | uncharacterized LOC100527528 |
| *Glyma.10G029500* | Chr10:2566905-2571623 | ETO1-like protein 1 |
| *Glyma.10G029600* | Chr10:2575885-2578579 | heat stress transcription factor Hsf-21 |
| *Glyma.10G031600* | Chr10:2754135-2759010 | transcription factor UNE12 |
| *Glyma.10G034300* | Chr10:2948272-2950521 | transcription factor bHLH3 |
| *Glyma.10G034400* | Chr10:2981775-3003608 | myosin-9 |
